# Clinical sepsis phenotypes in critically ill COVID-19 patients

**DOI:** 10.1186/s13054-022-04118-6

**Published:** 2022-08-09

**Authors:** Niklas Bruse, Emma J. Kooistra, Aron Jansen, Rombout B. E. van Amstel, Nicolette F. de Keizer, Jason N. Kennedy, Christopher Seymour, Lonneke A. van Vught, Peter Pickkers, Matthijs Kox

**Affiliations:** 1 Department of Intensive Care Medicine, Radboud University Medical Center, Postbus 9101, 6500 HB Nijmegen, The Netherlands; 2 Department of Intensive Care Medicine, Amsterdam UMC Location University of Amsterdam, Amsterdam, Netherlands; 3 Department of Medical Informatics, Amsterdam UMC Location University of Amsterdam, Amsterdam, The Netherlands; 4 Quality of Care & Methodology, Amsterdam Public Health, Amsterdam, The Netherlands; 5 National Intensive Care Evaluation (NICE) Foundation, Amsterdam, The Netherlands; 6 Department of Critical Care Medicine, School of Medicine, University of Pittsburgh, Pittsburgh, PA USA

**Keywords:** COVID-19, Phenotypes, Personalized medicine, Sepsis, Dexamethasone

## Abstract

**Background:**

A greater understanding of disease heterogeneity may facilitate precision medicine for coronavirus disease 2019 (COVID-19). Previous work identified four distinct clinical phenotypes associated with outcome and treatment responses in non-COVID-19 sepsis patients, but it is unknown if and how these phenotypes are recapitulated in COVID-19 sepsis patients.

**Methods:**

We applied the four non-COVID-19 sepsis phenotypes to a total of 52,274 critically ill patients, comprising two cohorts of COVID-19 sepsis patients (admitted before and after the introduction of dexamethasone as standard treatment) and three non-COVID-19 sepsis cohorts (non-COVID-19 viral pneumonia sepsis, bacterial pneumonia sepsis, and bacterial sepsis of non-pulmonary origin). Differences in proportions of phenotypes and their associated mortality were determined across these cohorts.

**Results:**

Phenotype distribution was highly similar between COVID-19 and non-COVID-19 viral pneumonia sepsis cohorts, whereas the proportion of patients with the δ-phenotype was greater in both bacterial sepsis cohorts compared to the viral sepsis cohorts. The introduction of dexamethasone treatment was associated with an increased proportion of patients with the δ-phenotype (6% vs. 11% in the pre- and post-dexamethasone COVID-19 cohorts, respectively, *p* < 0.001). Across the cohorts, the α-phenotype was associated with the most favorable outcome, while the δ-phenotype was associated with the highest mortality. Survival of the δ-phenotype was markedly higher following the introduction of dexamethasone (60% vs 41%, *p* < 0.001), whereas no relevant differences in survival were observed for the other phenotypes among COVID-19 patients.

**Conclusions:**

Classification of critically ill COVID-19 patients into clinical phenotypes may aid prognostication, prediction of treatment efficacy, and facilitation of personalized medicine.

**Supplementary Information:**

The online version contains supplementary material available at 10.1186/s13054-022-04118-6.

## Introduction

Over the last decades, dozens of clinical trials have employed a one-size-fits-all approach for critically ill patients, especially for those with sepsis, but virtually all failed to demonstrate clinical benefit. Along the same lines, the current undifferentiated treatment approach for coronavirus disease 2019 (COVID-19) may be inadequate, as mainly patients with high levels of inflammatory markers appear to benefit from anti-inflammatory medication[1, 2]. Consequently, personalized treatment may be warranted for which a better understanding of disease heterogeneity is pivotal[3]. Phenotyping is a method that is increasingly used to gain a deeper understanding of disease heterogeneity and may inform treatment responses. In non-COVID-19 sepsis patients, four clinical phenotypes with differential outcomes and treatment responses were previously identified[4]. Because COVID-19 fulfills the sepsis criteria, applying phenotypes previously derived in sepsis patients could represent the first step toward precision medicine for these patients. Therefore, we applied these phenotypes to critically ill COVID-19 sepsis patients admitted before and after the introduction of dexamethasone as standard treatment, and to three non-COVID-19 sepsis cohorts. We determined differences in proportions of phenotypes, their relationship with clinical outcome, and explored treatment responses.

## Methods

Detailed methods are provided in the Additional file 1. Data of 56,003 patients admitted to 82 Dutch Intensive Care Units (ICUs) from January 2016 to November 2021 were extracted from the national ICU registry (Nationale Intensive Care Evaluatie, NICE[5]). Patients were assigned to the following cohorts based on their primary diagnosis: COVID-19 pre-dexamethasone: January–April 2020; COVID-19 post-dexamethasone: September 2020–mid-November 2021; non-COVID-19 viral pneumonia sepsis: January 2016–September 2019; bacterial pneumonia sepsis: January 2016–September 2019; bacterial sepsis of non-pulmonary origin: January 2016–September 2019. The NICE dataset contained 17 of the 29 cluster variables used in the original phenotyping model [4], measured within the first 24 h of ICU admission, which are underlined in Table 1. The percentage missingness of cluster variables is described in Additional file 1: Table 1. After excluding patients with more than four missing cluster variables, 52,274 patients were used for analysis. Log-transformation, scaling, and centering were performed using the same approach as previously described for sepsis phenotype validation in external cohorts [4]. Subsequently, patients were mapped to the previously derived and validated centroids of the sepsis phenotypes by Euclidean distance [4]. All analyses were performed using R 3.6.1.

**Table 1:** Patient characteristics and outcomes

Parameters obtained within 24 h of ICU admission	COVID-19 pre-dexamethasone (n = 2288)	COVID-19 post-dexamethasone (n = 8596)	non-COVID-19 viral pneumonia (n = 3460)	Bacterial pneumonia (n = 19,947)	Sepsis of non-pulmonary origin (n = 17,983)
	Jan 2020–Apr 2020	Sep 2020–Nov 2021	Jan 2016–Sep 2019	Jan 2016–Sep 2019	Jan 2016–Sep 2019
Sex, male	1672 (73%)	5914 (69%)***	1823 (53%)***	12,147 (61%)***	10,373 (58%)***
BMI, kg/m^2^	27.8 [25.4—31.2]	29.2 [26.1—33.0]***	25.7 [22.6—29.7]***	25.2 [22.4—29.1]***	26.2 [23.2—30.3]***
Normal (< 25)	490 (21%)	1487 (17%)***	1516 (44%)***	9262 (46%)***	7037 (39%)***
Overweight (25 to < 30)	1032 (45%)	3281 (38%)***	1012 (29%)***	5957 (30%)***	5699 (32%)***
Obese Class 1: (30 to < 35)	481 (21%)	2261 (26%)***	464 (13%)***	2406 (12%)***	2631 (15%)***
Class 2: (35 to < 40)	161 (7%)	951 (11%)***	203 (6%)	947 (5%)***	1082 (6%)
Class 3: (> 40)	89 (4%)	508 (6%)***	148 (4%)	636 (3%)	821 (5%)
Age, years	65 [56—72]	64 [55—71]*	66 [57—74]***	69 [60—77]***	69 [59—77]***
APACHE IV score	58 [47—71]	59 [49—71]	64 [51—80]***	73 [58—91]***	78 [62—98]***
APACHE IV APS^a^ score	46 [38—57]	48 [40—58]*	50 [39—65]***	57 [44—74]***	62 [47—81]***
Aids	1 (0%)	7 (0%)	6 (0%)	87 (0%)*	42 (0%)
Cardiovascular insuffiency	23 (1%)	117 (1%)	115 (3%)***	877 (4%)***	754 (4%)***
Chronic dialysis	3 (0%)	53 (1%)*	33 (1%)***	244 (1%)***	471 (3%)***
Chronic renal insuffiency	60 (3%)	377 (4%)***	237 (7%)***	1769 (9%)***	2389 (13%)***
Cirrhosis	3 (0%)	40 (0%)	22 (1%)*	286 (1%)***	542 (3%)***
COPD	183 (8%)	809 (9%)	1551 (45%)***	6605 (33%)***	2248 (13%)***
Diabetes mellitus	435 (19%)	2050 (24%)***	693 (20%)	4186 (21%)	4696 (26%)***
Hematological malignancy	35 (2%)	138 (2%)	180 (5%)***	1030 (5%)***	995 (6%)***
Immunological insufficiency	167 (7%)	776 (9%)*	640 (18%)***	3683 (18%)***	3467 (19%)***
Metastatic neoplasm	16 (1%)	55 (1%)	68 (2%)***	1054 (5%)***	1280 (7%)***
Respiratory insufficiency	92 (4%)	361 (4%)	594 (17%)***	2336 (12%)***	581 (3%)
Comorbidity index^b^	0.44 (0.014)	0.55 (0.008)***	0.88 (0.014)***	0.84 (0.006)***	0.70 (0.007)***
Mechanical ventilation	1848 (81%)	5030 (59%)***	2449 (71%)***	11,321 (57%)***	5323 (30%)***
PaO2 (mmHg)	77 (66—94)	70 (60—83)***	74 (64—89)***	75 (64—90)***	82 (70—99)***
PaO_2_/FiO_2_ ratio	125 [90—173]	90 [69—124]***	167 [112—233]***	149 [98—221]***	257 [164—344]***
No ARDS (> 300 mmHg)	100 (4%)	176 (2%)***	316 (9%)***	1657 (8%)***	5352 (30%)***
Mild ARDS (> 200–≤ 300 mmHg)	238 (10%)	372 (4%)***	792 (23%)***	3617 (18%)***	4053 (23%)***
Moderate ARDS (> 100–≤ 200 mmHg)	1058 (46%)	2575 (30%)***	1315 (38%)***	7216 (36%)***	3647 (20%)***
Severe ARDS (≤ 100 mmHg)	684 (30%)	4645 (54%)***	623 (18%)***	4512 (23%)***	1366 (8%)***
PaCO_2_, mmHg	41 [35—48]	36 [32—43]***	47 [37—60]***	41 [33—52]	34 [29—40]***
Respiratory rate (max), breaths/min	31 [26—38]	33 [28—39]***	32 [27—39]***	32 [27—38]***	30 [25—35]***
Vasoactive medication	1569 (69%)	3945 (46%)***	1541 (45%)***	9872 (49%)***	12,140 (68%)
Hematocrit (min)	0.37 [0.34—0.39]	0.38 [0.35—0.41]***	0.37 [0.32—0.41]	0.34 [0.29—0.38]***	0.31 [0.27—0.35]***
Heart rate (max), beats/min	103 [91—116]	98 [87—112]***	116 [101—132]***	118 [102—135]***	116 [100—135]***
Mean arterial pressure (min), mmHg	62 [57—68]	66 [59—73]***	61 [54—69]**	59 [52—67]***	55 [48—62]***
Mean arterial pressure (max), mmHg	107 [97—121]	108 [98—121]	106 [94—121]**	100 [89—114]***	94 [84—106]***
Acute renal failure	204 (9%)	493 (6%)***	402 (12%)**	3650 (18%)***	6283 (35%)***
Creatinine, max, µmol/L	78 [63—102]	73 [60—98]**	84 [60—127]***	97 [67—156]***	146 [93—237]***
Blood urea nitrogen, mg/dL	18 [13—25]	23 [17—31]***	23 [15—35]***	28 [18—43]***	35 [22—54]***
Urinary output, L	1.20 [0.82—1.72]	1.60 [1.19—2.20]***	1.53 [1.00—2.23]***	1.48 [0.92—2.23]***	1.35 [0.66—2.20]***
Bilirubin, µmol/L	9 [6—12]	8 [6—12]***	8 [5—12]***	10 [6—16]***	14 [8—27]***
Sodium, max, mmol/L	138 [136—141]	139 [137—141]***	139 [136—142]***	139 [136—142]***	139 [136—142]***
Potassium (max), mmol/L	4.1 [3.8—4.4]	4.3 [4.0—4.6]***	4.4 [4.0—4.8]***	4.3 [4.0—4.8]***	4.3 [4.0—4.9]***
Glucose (max), mmol/L	8.4 [7.1—10.9]	11.5 [9.2—15.2]***	9.9 [7.9—12.5]***	9.5 [7.5—12.5]***	9.0 [7.1—12.1]***
pH (min)	7.39 [7.32—7.45]	7.44 [7.38—7.48]***	7.36 [7.29—7.43]***	7.38 [7.29—7.44]***	7.38 [7.30—7.44]***
Bicarbonate (max), mmol/L	26 [24—28]	26 [24—28]	28 [24—32]***	25 [22—29]***	22 [19—24]***
Albumin (min), g/L	26 [23—30]	28 [25—32]***	29 [25—34]***	26 [22—31]	24 [20—28]***
White blood cell count (max), × 10^9^/L	9.1 [6.9—12.0]	9.9 [7.2—13.2]***	11.0 [7.5—15.3]***	13.8 [9.4—19.6]***	15.9 [10.2—23.4]***
Thrombocytes (min), × 10^9^/L	228 [172—296]	240 [185—305]***	198 [143—261]***	208 [148—285]***	169 [104—254]***
Temperature, °C	38.7 [38.0—39.4]	37.5 [37.0—38.2]***	38.1 [37.4—38.8]***	38.0 [37.4—38.8]***	38.0 [37.3—38.9]***
**Outcome parameters**					
ICU length-of-stay survivors, days	15 [9—29]	8 [4—17]***	4 [2—8]***	3 [2—7]***	2 [1–5]***
ICU length-of-stay non-survivors, days	11 [5—21]	16 [8—25]***	6 [2—12]***	3 [1—8]***	2 [1–5]***
ICU mortality	609 (27%)	1965 (23%)***	583 (17%)***	3831 (19%)***	3250 (18%)***
Hospital length-of-stay survivors, days	29 [18—45]	19 [12—33]***	11 [7—20]***	13 [8—22]***	13 [7—24]***
Hospital length-of-stay non-survivors, days	15 [8—24]	20 [12—30]***	9 [4—17]***	8 [3—16]***	7 [2—17]***
In-hospital mortality	666 (29%)	2181 (25%)**	755 (22%)***	5257 (26%)*	4456 (25%)***
28-day in-hospital mortality	554 (24%)	1588 (18%)***	676 (20%)***	4705 (24%)	3932 (22%)
90-day in-hospital mortality	664 (29%)	2168 (25%)**	753 (22%)***	5228 (26%)*	4407 (25%)***

Underlined parameters were used for clustering. Data are presented as median [interquartile range], mean (standard error of the mean), or number (%). * indicates p = 0.01 -0.05, ** indicates p = 0.001–0.01, *** indicates p = 0–0.001 compared with the COVID-19 pre-dexamethasone cohort, calculated using Dunn’s post hoc tests (following Kruskal–Wallis tests across all cohorts which all yielded *p* < 0.05) or Bonferroni-corrected pairwise chi-square tests (following chi-square tests across all cohorts which all yielded *p* < 0.05). ^a^Acute physiology score. ^b^Calculated by adding one point for each of the following comorbidities present: AIDS, cardiovascular insufficiency, chronic dialysis, chronic renal insufficiency, cirrhosis, COPD or respiratory insufficiency, diabetes mellitus, hematologic malignancy, immune insufficiency, and metastatic neoplasm. AIDS: acquired immunodeficiency syndrome, APACHE IV: Acute Physiology and Chronic Health Evaluation IV, COVID-19: coronavirus disease 2019, BMI: body mass index, COPD: chronic obstructive pulmonary disease, ARDS: acute respiratory distress syndrome, ICU: intensive care unit

## Results

**Fig. 1:**
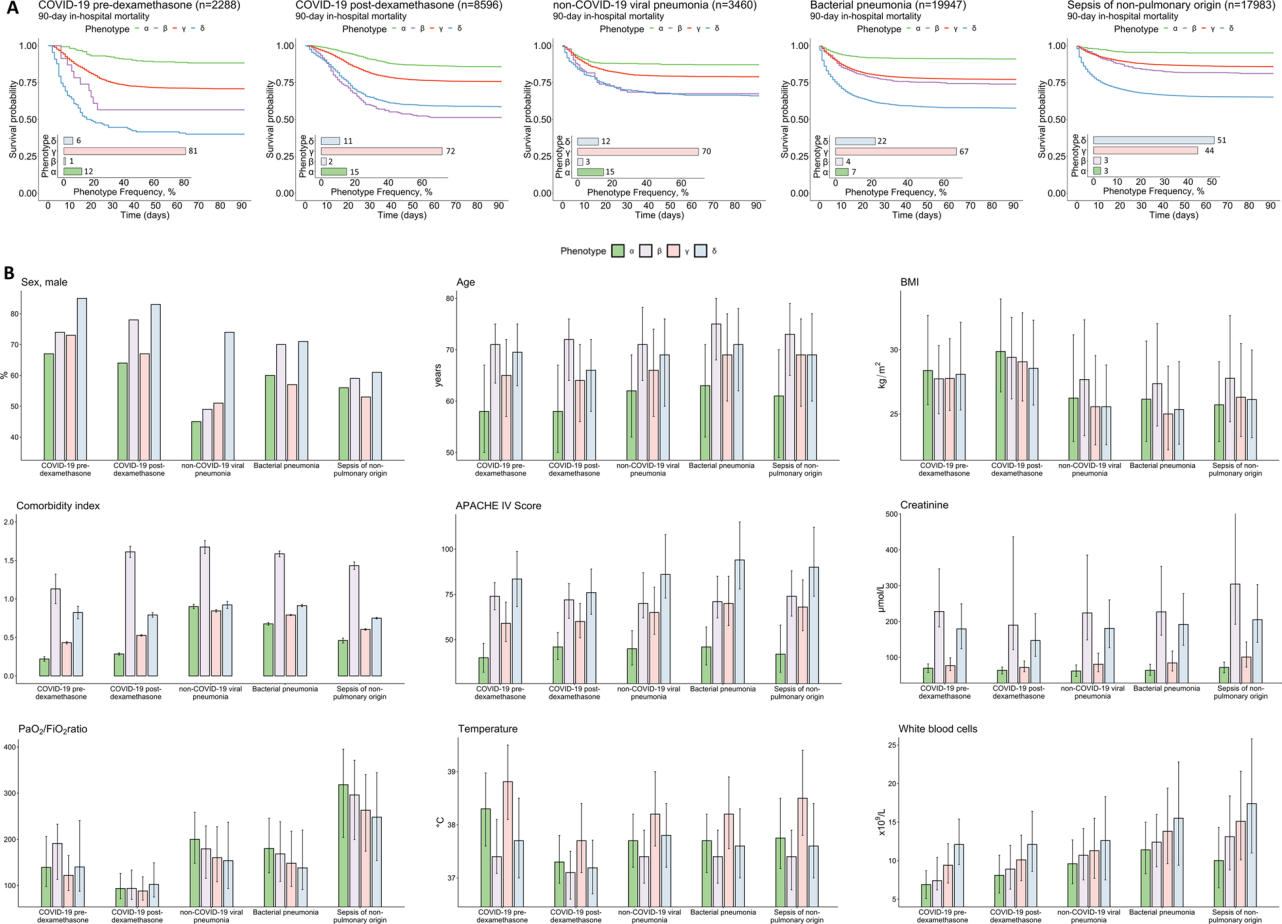
**Distribution, characteristics, and outcome of phenotypes in patients with COVID-19 sepsis and sepsis of other origins**. **A** Phenotype distribution and 90-day in-hospital mortality Kaplan–Meier curves for patients in the COVID-19 pre-dexamethasone cohort, COVID-19 post-dexamethasone cohort, non-COVID-19 viral pneumonia sepsis cohort, bacterial pneumonia sepsis cohort, and bacterial sepsis of non-pulmonary origin cohort. **B** Sex, age, body mass index (BMI), comorbidity index, Acute Physiology and Chronic Health Evaluation (APACHE) IV score, creatinine, P_a_O_2_/FiO_2_ ratio, temperature, and white blood cell count for each phenotype across all above-mentioned cohorts. Data in panel B are shown as percentage (sex), mean and standard error of the mean (comorbidity index) or median and interquartile range (all other variables)

The proportion of patients with the β-phenotype was small in all cohorts, ranging from 1 to 4% (Fig. 1A), which limits the interpretation of this particular phenotype. Phenotype distribution was very similar between the COVID-19 and non-COVID-19 viral pneumonia sepsis cohorts. In both bacterial sepsis cohorts (pulmonary and non-pulmonary), the proportion of patients with the δ-phenotype was greater than in the viral sepsis cohorts at the expense of the α- and, especially, the γ-phenotype. This was particularly apparent for patients with bacterial sepsis of non-pulmonary origin. The latter was also the only cohort where the proportion of the δ-phenotype was greater than that of the γ-phenotype. In COVID-19 patients, the introduction of dexamethasone as standard treatment was associated with an increased proportion of patients with the δ-phenotype (6% vs. 11% in the pre- and post-dexamethasone cohorts, respectively, *p* < 0.001), at the expense of the γ-phenotype (81% versus 72%, *p* < 0.001).

Characteristics of all five cohorts are described in Table 1, Fig. 1B, and Additional file 1: Tables 2–6. Disregarding the nonabundant β-phenotype, patients with the α-phenotype were the youngest, had the highest BMI, and had relatively few comorbidities across all cohorts. This phenotype also presented with the lowest creatinine levels and white blood cell counts, and the highest PaO_2_/FiO_2_ ratios. This translated into the lowest APACHE IV scores of all phenotypes in all cohorts. Overall, the δ-phenotype exhibited the greatest proportion of males as well as the oldest patients with most comorbidities, highest creatinine levels and white blood cell counts, and therefore the highest APACHE IV scores. Representing the majority of the patient population in most cohorts, the γ-phenotype falls in between the extremes of the above-described phenotypes, only displaying the highest temperature across all cohorts.

Outcome differences between phenotypes were most pronounced for the pre-dexamethasone COVID-19 cohort and least pronounced in the non-COVID-19 viral pneumonia sepsis cohort (Fig. 1A). In all cohorts, the α-phenotype displayed the lowest 90-day mortality, while patients with the δ-phenotype generally displayed the highest mortality. Of interest, survival of the δ-phenotype was markedly higher following the introduction of dexamethasone as standard therapy for COVID-19 (60% vs 41%, *p* < 0.001, Fig. 1A), whereas no relevant differences in survival were observed for the other phenotypes among COVID-19 patients. Furthermore, across all phenotypes, hospital length-of-stay was 10 days shorter among survivors following the introduction of dexamethasone for COVID-19 patients, whereas the opposite was the case for non-survivors (5 days longer, see Table 1). Hospital length-of-stay also clearly varied between phenotypes in the different cohorts. For survivors, the α-phenotype exhibited the shortest (22 [14–33], 16 [11–27], 9 [6–14], 9 [5–14], and 7 [4–14] days for the pre-dexamethasone, post-dexamethasone, non-COVID-19 viral pneumonia sepsis, bacterial pneumonia sepsis, and bacterial sepsis of non-pulmonary origin cohorts, respectively) and the δ-phenotype the most prolonged length-of-stay (34 [24–57], 23 [13–42], 15 [9–30], 15 [8–26], and 14 [8–25] days, respectively) across all cohorts. For non-surviving COVID-19 patients, there were clear differences in length-of-stay between phenotypes, with the α-phenotype displaying the most prolonged (pre-dexamethasone: 18 [14–29] days, post-dexamethasone: 25 [17–36] days) and the δ-phenotype the shortest length-of-stay (9 [5–16] and 17 [10–26] days). A similar pattern was observed for patients with sepsis of non-pulmonary origin (α: 11 [5–17] days, δ: 5 [2–13] days), whereas no major differences in length-of-stay of non-survivors between phenotypes were observed for the other sepsis cohorts (data not shown).

## Discussion

We applied previously established clinical sepsis phenotypes[4] to 52,274 critically ill patients with COVID-19 or sepsis of other etiologies. Overall, the characteristics and gradation in mortality associated with the phenotypes were comparable across the cohorts and also similar to those reported in the original work[4], with the α-phenotype showing the most favorable outcome, whereas the δ-phenotype was associated with the highest mortality. However, compared to the previous study in non-COVID-19 sepsis patients[4], our non-COVID-19 sepsis cohorts contained very small proportions of patients with the β-phenotype as well as relatively low proportions of patients with the α-phenotype and greater predominance of the γ-phenotype. Because the characteristics of the different phenotypes are similar between our (bacterial) sepsis cohorts and those in the original investigation[4], these different proportions are likely due to differences in the overall composition of these cohorts between the studies. For instance, there are marked differences in sex, WBC counts, and glucose concentration. Furthermore, differences in ICU admission criteria between the Netherlands and the countries from which data were obtained in the original study [4] may be involved, such as the presence of multiple comorbidities.

Of interest, following the introduction of dexamethasone treatment as standard therapy for COVID-19, a significantly higher proportion of COVID-19 patients admitted to the ICU exhibited the δ-phenotype, while at the same time, survival of this phenotype was better compared to the pre-dexamethasone cohort. Factors such as differences in virulence or host response to later variants could be involved in the increased proportion of patients with the δ-phenotype. Furthermore, the use of dexamethasone (which was often already started on the ward prior to ICU admission) may have led to a shift in phenotype by itself or resulted in a slightly different patient population ultimately requiring ICU care. The use of dexamethasone (and later also tocilizumab) may have contributed to the improved outcome of the δ-phenotype, because it is associated with the highest white blood cell counts in our COVID-19 cohort and was previously shown to be characterized by the most elevated IL-6 and TNF levels in sepsis patients[4]. Interestingly, the introduction of dexamethasone treatment also appears to impact length-of-stay for both surviving and non-surviving COVID-19 patients. For all phenotypes, the length-of-stay for survivors was much shorter after the introduction of dexamethasone as standard treatment, while length-of-stay for non-survivors was longer. As the majority of patients in our cohort survived, this observation supports the positive effects of immunomodulatory treatment for COVID-19, because overall, patients spent less time in the ICU.

This work has several limitations. First, related to the observational nature, other improvements in patient management during the course of the pandemic, such as alterations in ventilation and anticoagulation strategies, may be involved in better survival and changes in length-of-stay of the post-dexamethasone cohort as well. Nevertheless, the finding that especially patients with a hyperinflamed phenotype demonstrated better outcome following the introduction of dexamethasone is a plausible one, but in view of multiple changes in other covariates over time, this requires validation. Second, only 17 of the 29 cluster variables used in the original work[4] were available in the NICE database. However, the phenotypes were successfully validated before in cohorts with similar numbers of missing variables [4]. Third, individual patient data on applied treatments (e.g., dexamethasone) as well as causative pathogens or SARS-CoV-2 variants were not available.

In conclusion, this work underlines that classification of critically ill COVID-19 patients into clinical phenotypes may aid prognostication, prediction of treatment efficacy, and facilitation of personalized medicine.

## Supplementary Information

**Additional file 1.** Online Supplement containing Supplementary Methods and Supplementary Table 1-6.

## Data Availability

Not applicable.

## Abbreviations

COVID-19: Coronavirus disease 2019
ICUs: Intensive care units
NICE: National ICU Registry (Nationale Intensive Care Evaluatie)
SARS-CoV-2: Severe acute respiratory syndrome coronavirus type 2
