# Supplementary material for: Clinical sepsis phenotypes in critically ill COVID-19 patients
Source: Crit Care. 2022 Aug 9;26:244. doi: 10.1186/s13054-022-04118-6 (PMC9361232; doi:10.1186/s13054-022-04118-6)
Supplement: Supplementary file 1 — Additional file 1. Online Supplement containing Supplementary Methods and Supplementary Table 1-6. [file 13054_2022_4118_MOESM1_ESM.docx]

**Online Supplement ‘Clinical sepsis phenotypes in critically ill COVID-19 patients’**

**Supplementary Methods**

Data collection in NICE is standardized using strict definitions and subject to data quality checks. All collected data were anonymized by NICE, and, in accordance with Dutch legislation and compliant with the European General Data Protection Regulation, the need to obtain consent was therefore waived. For the COVID-19 cohorts, only patients with severe acute respiratory syndrome coronavirus type 2 (SARS-CoV-2) infection as primary admission diagnosis were included. The primary diagnoses for the other cohorts were: non-COVID-19 viral pneumonia sepsis: ‘viral pneumonia’; bacterial pneumonia sepsis: ‘bacterial pneumonia’ and ‘pulmonary sepsis’; bacterial sepsis of non-pulmonary origin: ‘cutaneous/soft tissue sepsis’, ‘gastrointestinal sepsis’, ‘gynecologic sepsis’, ‘other sepsis’, ‘renal/urinary tract infection sepsis (including bladder)’, ‘other sepsis’, ‘septic arthritis’, and ‘cellulitis and localized soft tissue infections’. The period spanning May to August 2020 was used as a ‘washout period’ between the pre-dexamethasone and post-dexamethasone periods. During this period, very few COVID-19 patients were admitted to the ICU due to low numbers of infections in this summer period in the Netherlands (see <https://www.stichting-nice.nl/covid-19-op-de-ic.jsp>). For patients who were readmitted to the ICU during the same hospitalization period only data of the first ICU admission were used for clustering and analysis of baseline characteristics. Variables were filtered for missing values and for values that were out of range according to the NICE data dictionary. As described in the main manuscript, patients with more than four missing cluster variables were excluded, leaving 52,274 patients for analysis (pre-dexamethasone: n=2288, post-dexamethasone: n=8595, non-COVID-19 viral pneumonia sepsis: n=3460, bacterial pneumonia sepsis: n=19,947, bacterial sepsis of non-pulmonary origin: n=17,983). Percentages of missing cluster variables for included patients are listed in Supplementary Table 1. For these patients, missing data were imputed using multiple imputation with chained equations with the missRanger R package which produces a single new dataset. If patients were transferred between hospitals, periods of stay were added up to calculate the total length-of-stay.

**Supplementary Table 1: Percentage missingness of cluster variables per cohort.**

|  | **COVID-19  pre-dexamethasone (n=2288)**  **Jan 2020 – Apr 2020** | **COVID-19 post-dexamethasone  (n=8596)**  **Sep 2020 – Nov 2021** | **non-COVID-19 viral  pneumonia**  **(n=3460)**  **Jan 2016 – Sep 2019** | **Bacterial**  **pneumonia**  **(n=19947)**  **Jan 2016 – Sep 2019** | **Sepsis of  non-pulmonary  origin (n=17983)**  **Jan 2016 – Sep 2019** |
| --- | --- | --- | --- | --- | --- |
| Sex, male | 0 | 0 | 0 | 0 | 0 |
| Age, years | 0 | 0 | 0 | 0 | 0 |
| Comorbidity index^b^ | 0 | 0 | 0 | 0 | 0 |
| PaO_2_ (mmHg) | 8 | 7 | 10 | 12 | 17 |
| Respiratory rate (max), breaths/min | 1 | 1 | 1 | 1 | 1 |
| Heart rate (max), beats/min | 0 | 0 | 0 | 0 | 0 |
| Mean arterial pressure (max), mmHg | 0 | 0 | 0 | 1 | 1 |
| Creatinine, max, µmol/L | 0 | 0 | 1 | 0 | 1 |
| Blood urea nitrogen, mg/dL | 3 | 6 | 5 | 4 | 5 |
| Bilirubin, µmol/L | 13 | 13 | 23 | 23 | 19 |
| Sodium, max, mmol/L | 1 | 1 | 0 | 0 | 0 |
| Glucose (max), mmol/L | 2 | 2 | 1 | 2 | 2 |
| Bicarbonate (max), mmol/L | 2 | 7 | 3 | 4 | 7 |
| Albumin (min), g/L | 20 | 20 | 26 | 26 | 25 |
| White blood cell count (max), x10^9^/L | 1 | 1 | 1 | 1 | 1 |
| Thrombocytes (min), x10^9^/L | 1 | 1 | 1 | 2 | 2 |
| Temperature, °C | 1 | 1 | 1 | 1 | 1 |

**Supplementary Table 2: Patient characteristics and outcomes of the pre-dexamethasone cohort.**

| **Parameters obtained within 24 hours of ICU admission** | **All (n=2288)** | **Alpha (n=273, 12%)** | **Beta (n=23, 1%)** | **Gamma (n=1862, 81%)** | **Delta (n=130, 6%)** |
| --- | --- | --- | --- | --- | --- |
| Sex, male | 1672 (73%) | 182 (67%) | 17 (74%) | 1363 (73%) | 110 (85%) |
| BMI, kg/m^2^ | 27.8 [25.4 - 31.2] | 28.4 [25.7 - 32.7] | 27.7 [25.0 - 30.3] | 27.8 [25.3 - 31.0] | 28.1 [25.3 - 32.1] |
| Normal (<25) | 490 (21%) | 45 (16%) | 6 (26%) | 413 (22%) | 26 (20%) |
| Overweight (25 to <30) | 1032 (45%) | 115 (42%) | 10 (43%) | 849 (46%) | 58 (45%) |
| Obese Class 1: (30 to < 35) | 481 (21%) | 64 (23%) | 5 (22%) | 380 (20%) | 32 (25%) |
| Class 2: (35 to < 40) | 161 (7%) | 26 (10%) | 2 (9%) | 125 (7%) | 8 (6%) |
| Class 3: (>40) | 89 (4%) | 17 (6%) | 0 (0%) | 67 (4%) | 5 (4%) |
| Age, years | 65 [56 - 72] | 58 [50 - 67] | 71 [64 - 75] | 65 [57 - 72] | 70 [63 - 75] |
| APACHE IV score | 58 [47 - 71] | 40 [32 - 48] | 74 [66 - 82] | 59 [49 - 71] | 84 [68 - 99] |
| APACHE IV APS^a^ score | 46 [38 - 57] | 31 [25 - 38] | 59 [45 - 65] | 47 [39 - 57] | 68 [56 - 85] |
| Aids | 1 (0%) | 0 (0%) | 0 (0%) | 1 (0%) | 0 (0%) |
| Cardiovascular insuffiency | 23 (1%) | 2 (1%) | 0 (0%) | 11 (1%) | 10 (8%) |
| Chronic dialysis | 3 (0%) | 0 (0%) | 1 (4%) | 0 (0%) | 2 (2%) |
| Chronic renal insuffiency | 60 (3%) | 1 (0%) | 10 (43%) | 30 (2%) | 19 (15%) |
| Cirrhosis | 3 (0%) | 0 (0%) | 0 (0%) | 2 (0%) | 1 (1%) |
| COPD | 183 (8%) | 15 (5%) | 2 (9%) | 160 (9%) | 6 (5%) |
| Diabetes mellitus | 435 (19%) | 21 (8%) | 7 (30%) | 362 (19%) | 45 (35%) |
| Hematological malignancy | 35 (2%) | 2 (1%) | 0 (0%) | 31 (2%) | 2 (2%) |
| Immunological insufficiency | 167 (7%) | 9 (3%) | 4 (17%) | 141 (8%) | 13 (10%) |
| Metastatic neoplasm | 16 (1%) | 0 (0%) | 0 (0%) | 13 (1%) | 3 (2%) |
| Respiratory insufficiency | 92 (4%) | 10 (4%) | 3 (13%) | 73 (4%) | 6 (5%) |
| Comorbidity index^b^ | 0.44 (0.014) | 0.22 (0.030) | 1.13 (0.192) | 0.43 (0.015) | 0.82 (0.081) |
| Mechanical ventilation | 1848 (81%) | 185 (68%) | 20 (87%) | 1527 (82%) | 116 (89%) |
| PaO_2_ (mmHg) | 77 (66 - 94) | 76 (65 - 98) | 90 (73 - 111) | 86 (71 - 116) | 77 (66 - 92) |
| PaO_2_/FiO_2_ ratio | 125 [90 - 173] | 139 [98 - 206] | 191 [113 - 233] | 122 [89 - 165] | 140 [87 - 241] |
| No ARDS (>300 mmHg) | 100 (4%) | 20 (7%) | 2 (9%) | 58 (3%) | 20 (15%) |
| Mild ARDS (>200 - ≤300 mmHg) | 238 (10%) | 44 (16%) | 4 (17%) | 170 (9%) | 20 (15%) |
| Moderate ARDS (>100 - ≤200 mmHg) | 1058 (46%) | 110 (40%) | 8 (35%) | 900 (48%) | 40 (31%) |
| Severe ARDS (≤100 mmHg) | 684 (30%) | 64 (23%) | 2 (9%) | 575 (31%) | 43 (33%) |
| PaCO_2_, mmHg | 41 [35 - 48] | 39 [34 - 43] | 44 [39 - 49] | 41 [35 - 48] | 42 [34 - 49] |
| Respiratory rate (max), breaths/min | 31 [26 - 38] | 25 [22 - 28] | 25 [21 - 29] | 32 [27 - 39] | 30 [26 - 38] |
| Vasoactive medication | 1569 (69%) | 143 (52%) | 20 (87%) | 1296 (70%) | 110 (85%) |
| Hematocrit (min) | 0.37 [0.34 - 0.39] | 0.37 [0.34 - 0.40] | 0.34 [0.28 - 0.36] | 0.37 [0.34 - 0.39] | 0.37 [0.32 - 0.39] |
| Heart rate (max), beats/min | 103 [91 - 116] | 90 [81 - 100] | 86 [76 - 95] | 105 [94 - 118] | 110 [95 - 126] |
| Mean arterial pressure (min), mmHg | 62 [57 - 68] | 66 [61 - 74] | 63 [60 - 68] | 62 [57 - 68] | 57 [50 - 64] |
| Mean arterial pressure (max), mmHg | 107 [97 - 121] | 106 [96 - 115] | 100 [90 - 110] | 108 [97 - 122] | 100 [92 - 115] |
| Acute renal failure | 204 (9%) | 2 (1%) | 11 (48%) | 133 (7%) | 58 (45%) |
| Creatinine, max, µmol/L | 78 [63 - 102] | 70 [58 - 82] | 228 [185 - 347] | 77 [63 - 99] | 180 [124 - 250] |
| Blood urea nitrogen, mg/dL | 18 [13 - 25] | 13 [10 - 17] | 51 [41 - 63] | 18 [13 - 24] | 44 [28 - 62] |
| Urinary output, L | 1.20 [0.82 - 1.72] | 1.30 [0.90 - 2.00] | 0.90 [0.39 - 1.10] | 1.20 [0.86 - 1.70] | 0.80 [0.49 - 1.38] |
| Bilirubin, µmol/L | 9 [6 - 12] | 8 [6 - 10] | 5 [4 - 7] | 9 [6 - 12] | 13 [9 - 20] |
| Sodium, max, mmol/L | 138 [136 - 141] | 139 [136 - 141] | 140 [136 - 142] | 138 [136 - 141] | 141 [138 - 144] |
| Potassium (max), mmol/L | 4.1 [3.8 - 4.4] | 3.9 [3.7 - 4.2] | 4.3 [4.1 - 5.2] | 4.1 [3.8 - 4.4] | 4.5 [4.1 - 5.2] |
| Glucose (max), mmol/L | 8.4 [7.1 - 10.9] | 7.4 [6.4 - 8.7] | 10.0 [7.8 - 13.5] | 8.5 [7.2 - 10.9] | 10.9 [9.3 - 14.4] |
| pH (min) | 7.39 [7.32 - 7.45] | 7.42 [7.38 - 7.47] | 7.31 [7.24 - 7.38] | 7.39 [7.33 - 7.44] | 7.30 [7.24 - 7.37] |
| Bicarbonate (max), mmol/L | 26 [24 - 28] | 26 [25 - 28] | 23 [22 - 26] | 26 [24 - 28] | 23 [21 - 24] |
| Albumin (min), g/L | 26 [23 - 30] | 30 [28 - 33] | 27 [21 - 30] | 26 [22 - 29] | 26 [23 - 30] |
| White blood cell count (max), x10^9^/L | 9.1 [6.9 - 12.0] | 6.9 [5.1 - 8.7] | 7.4 [6.2 - 10.4] | 9.4 [7.1 - 12.2] | 12.1 [9.5 - 15.4] |
| Thrombocytes (min), x10^9^/L | 228 [172 - 296] | 206 [160 - 259] | 207 [163 - 268] | 233 [177 - 302] | 212 [144 - 269] |
| Temperature, °C | 38.7 [38.0 - 39.4] | 38.3 [37.6 - 39.0] | 37.4 [37.1 - 38.1] | 38.8 [38.1 - 39.5] | 37.7 [37.0 - 38.5] |
| **Outcome parameters** |  |  |  |  |  |
| ICU length-of-stay survivors, days | 15 [9 - 29] | 12 [4 - 19] | 19 [10 - 36] | 16 [9 - 30] | 20 [10 - 29] |
| ICU length-of-stay nonsurvivors, days | 11 [5 - 21] | 15 [9 - 27] | 13 [8 - 17] | 12 [6 - 22] | 6 [3 - 13] |
| ICU mortality | 609 (27%) | 24 (9%) | 10 (43%) | 505 (27%) | 70 (54%) |
| Hospital length-of-stay survivors, days | 29 [18 - 45] | 22 [15 - 34] | 47 [20 - 59] | 31 [19 - 45] | 34 [24 - 57] |
| Hospital length-of-stay nonsurvivors, days | 15 [8 - 24] | 18 [14 - 34] | 14 [10 - 20] | 16 [8 - 24] | 8 [5 - 15] |
| In-hospital mortality | 666 (29%) | 32 (12%) | 10 (43%) | 546 (29%) | 78 (60%) |
| 28-day in-hospital mortality | 554 (24%) | 20 (7%) | 10 (43%) | 452 (24%) | 72 (55%) |
| 90-day in-hospital mortality | 664 (29%) | 32 (12%) | 10 (43%) | 544 (29%) | 78 (60%) |

Underlined parameters were used for clustering. Data are presented as median [interquartile range], mean (standard error of the mean), or number (%). ^a^Acute physiology score. ^b^Calculated by adding one point for each of the following comorbidities present: AIDS, cardiovascular insufficiency, chronic dialysis, chronic renal insufficiency, cirrhosis, COPD or respiratory insufficiency, diabetes mellitus, hematologic malignancy, immune insufficiency, and metastatic neoplasm. AIDS: acquired immunodeficiency syndrome, APACHE IV: Acute Physiology and Chronic Health Evaluation IV, COVID-19: coronavirus disease 2019, BMI: body mass index, COPD: chronic obstructive pulmonary disease, ARDS: acute respiratory distress syndrome, ICU: intensive care unit.

**Supplementary Table 3: Patient characteristics and outcomes of the post-dexamethasone cohort.**

| **Parameters obtained within 24 hours of ICU admission** | **All (n=8596)** | **Alpha (n=1261, 15%)** | **Beta (n=216, 2%)** | **Gamma (n=6201, 72%)** | **Delta (n=918, 11%)** |
| --- | --- | --- | --- | --- | --- |
| Sex, male | 5914 (69%) | 808 (64%) | 169 (78%) | 4178 (67%) | 759 (83%) |
| BMI, kg/m^2^ | 29.2 [26.1 - 33.0] | 29.9 [26.7 - 34.0] | 29.4 [26.2 - 32.5] | 29.1 [26.0 - 32.9] | 28.6 [25.7 - 32.3] |
| Normal (<25) | 1487 (17%) | 180 (14%) | 38 (18%) | 1069 (17%) | 200 (22%) |
| Overweight (25 to <30) | 3281 (38%) | 451 (36%) | 79 (37%) | 2399 (39%) | 352 (38%) |
| Obese Class 1: (30 to < 35) | 2261 (26%) | 354 (28%) | 66 (31%) | 1623 (26%) | 218 (24%) |
| Class 2: (35 to < 40) | 951 (11%) | 162 (13%) | 22 (10%) | 676 (11%) | 91 (10%) |
| Class 3: (>40) | 508 (6%) | 104 (8%) | 7 (3%) | 355 (6%) | 42 (5%) |
| Age, years | 64 [55 - 71] | 58 [50 - 67] | 72 [64 - 76] | 64 [56 - 71] | 66 [58 - 72] |
| APACHE IV score | 59 [49 - 71] | 46 [39 - 54] | 72 [62 - 81] | 60 [51 - 70] | 76 [64 - 89] |
| APACHE IV APS^a^ score | 48 [40 - 58] | 38 [31 - 44] | 56 [46 - 66] | 48 [41 - 57] | 63 [53 - 76] |
| Aids | 7 (0%) | 0 (0%) | 0 (0%) | 4 (0%) | 3 (0%) |
| Cardiovascular insuffiency | 117 (1%) | 6 (0%) | 13 (6%) | 78 (1%) | 20 (2%) |
| Chronic dialysis | 53 (1%) | 0 (0%) | 30 (14%) | 8 (0%) | 15 (2%) |
| Chronic renal insuffiency | 377 (4%) | 2 (0%) | 114 (53%) | 125 (2%) | 136 (15%) |
| Cirrhosis | 40 (0%) | 1 (0%) | 0 (0%) | 18 (0%) | 21 (2%) |
| COPD | 809 (9%) | 99 (8%) | 33 (15%) | 614 (10%) | 63 (7%) |
| Diabetes mellitus | 2050 (24%) | 136 (11%) | 93 (43%) | 1491 (24%) | 330 (36%) |
| Hematological malignancy | 138 (2%) | 7 (1%) | 3 (1%) | 110 (2%) | 18 (2%) |
| Immunological insufficiency | 776 (9%) | 61 (5%) | 48 (22%) | 576 (9%) | 91 (10%) |
| Metastatic neoplasm | 55 (1%) | 5 (0%) | 1 (0%) | 45 (1%) | 4 (0%) |
| Respiratory insufficiency | 361 (4%) | 47 (4%) | 18 (8%) | 264 (4%) | 32 (3%) |
| Comorbidity index^b^ | 0.55 (0.008) | 0.28 (0.015) | 1.61 (0.073) | 0.53 (0.009) | 0.79 (0.029) |
| Mechanical ventilation | 5030 (59%) | 612 (49%) | 123 (57%) | 3662 (59%) | 633 (69%) |
| PaO_2_ (mmHg) | 70 (60 - 83) | 70 (61 - 83) | 70 (60 - 84) | 77 (65 - 97) | 68 (60 - 82) |
| PaO_2_/FiO_2_ ratio | 90 [69 - 124] | 93 [71 - 126] | 94 [70 - 133] | 88 [68 - 119] | 102 [75 - 149] |
| No ARDS (>300 mmHg) | 176 (2%) | 32 (3%) | 7 (3%) | 84 (1%) | 53 (6%) |
| Mild ARDS (>200 - ≤300 mmHg) | 372 (4%) | 57 (5%) | 15 (7%) | 240 (4%) | 60 (7%) |
| Moderate ARDS (>100 - ≤200 mmHg) | 2575 (30%) | 377 (30%) | 57 (26%) | 1820 (29%) | 321 (35%) |
| Severe ARDS (≤100 mmHg) | 4645 (54%) | 629 (50%) | 105 (49%) | 3490 (56%) | 421 (46%) |
| PaCO_2_, mmHg | 36 [32 - 43] | 37 [34 - 43] | 35 [30 - 41] | 36 [32 - 43] | 36 [30 - 46] |
| Respiratory rate (max), breaths/min | 33 [28 - 39] | 28 [24 - 31] | 27 [24 - 31] | 34 [29 - 40] | 32 [28 - 38] |
| Vasoactive medication | 3945 (46%) | 426 (34%) | 95 (44%) | 2815 (45%) | 609 (66%) |
| Hematocrit (min) | 0.38 [0.35 - 0.41] | 0.39 [0.37 - 0.42] | 0.33 [0.29 - 0.37] | 0.38 [0.35 - 0.41] | 0.38 [0.34 - 0.41] |
| Heart rate (max), beats/min | 98 [87 - 112] | 87 [78 - 96] | 84 [74 - 94] | 101 [89 - 114] | 107 [92 - 123] |
| Mean arterial pressure (min), mmHg | 66 [59 - 73] | 70 [64 - 79] | 66 [60 - 73] | 66 [59 - 72] | 61 [53 - 67] |
| Mean arterial pressure (max), mmHg | 108 [98 - 121] | 107 [98 - 119] | 105 [92 - 120] | 109 [98 - 122] | 104 [94 - 119] |
| Acute renal failure | 493 (6%) | 4 (0%) | 66 (31%) | 201 (3%) | 222 (24%) |
| Creatinine, max, µmol/L | 73 [60 - 98] | 64 [54 - 73] | 190 [122 - 436] | 72 [60 - 90] | 147 [103 - 222] |
| Blood urea nitrogen, mg/dL | 23 [17 - 31] | 18 [14 - 23] | 58 [42 - 81] | 22 [17 - 29] | 43 [31 - 62] |
| Urinary output, L | 1.60 [1.19 - 2.20] | 1.70 [1.20 - 2.33] | 1.32 [0.80 - 2.02] | 1.60 [1.20 - 2.20] | 1.40 [0.90 - 2.05] |
| Bilirubin, µmol/L | 8 [6 - 12] | 8 [6 - 10] | 6 [4 - 8] | 8 [6 - 11] | 12 [8 - 18] |
| Sodium, max, mmol/L | 139 [137 - 141] | 140 [138 - 142] | 138 [136 - 141] | 139 [136 - 141] | 141 [138 - 144] |
| Potassium (max), mmol/L | 4.3 [4.0 - 4.6] | 4.2 [4.0 - 4.5] | 4.5 [4.2 - 5.0] | 4.3 [4.0 - 4.6] | 4.6 [4.2 - 5.1] |
| Glucose (max), mmol/L | 11.5 [9.2 - 15.2] | 10.1 [8.4 - 12.7] | 12.9 [9.8 - 16.1] | 11.5 [9.3 - 15.0] | 14.9 [11.4 - 19.3] |
| pH (min) | 7.44 [7.38 - 7.48] | 7.46 [7.41 - 7.49] | 7.40 [7.34 - 7.45] | 7.45 [7.39 - 7.49] | 7.36 [7.27 - 7.43] |
| Bicarbonate (max), mmol/L | 26 [24 - 28] | 27 [26 - 29] | 24 [21 - 26] | 26 [24 - 28] | 23 [20 - 25] |
| Albumin (min), g/L | 28 [25 - 32] | 33 [30 - 35] | 29 [25 - 32] | 28 [24 - 31] | 28 [24 - 31] |
| White blood cell count (max), x10^9^/L | 9.9 [7.2 - 13.2] | 8.1 [5.8 - 10.7] | 8.9 [6.3 - 12.0] | 10.1 [7.4 - 13.3] | 12.1 [8.6 - 16.4] |
| Thrombocytes (min), x10^9^/L | 240 [185 - 305] | 238 [192 - 298] | 218 [165 - 288] | 247 [191 - 312] | 200 [147 - 264] |
| Temperature, °C | 37.5 [37.0 - 38.2] | 37.3 [36.9 - 37.8] | 37.1 [36.6 - 37.5] | 37.7 [37.1 - 38.4] | 37.2 [36.7 - 37.7] |
| **Outcome parameters** |  |  |  |  |  |
| ICU length-of-stay survivors, days | 8 [4 - 17] | 7 [3 - 13] | 6 [2 - 22] | 9 [5 - 17] | 10 [4 - 24] |
| ICU length-of-stay nonsurvivors, days | 16 [8 - 25] | 22 [12 - 31] | 13 [7 - 22] | 16 [9 - 26] | 14 [7 - 21] |
| ICU mortality | 1965 (23%) | 162 (13%) | 89 (41%) | 1359 (22%) | 355 (39%) |
| Hospital length-of-stay survivors, days | 19 [12 - 33] | 16 [11 - 27] | 22 [14 - 42] | 19 [13 - 33] | 23 [13 - 44] |
| Hospital length-of-stay nonsurvivors, days | 20 [12 - 30] | 26 [16 - 36] | 16 [11 - 26] | 20 [13 - 30] | 17 [10 - 25] |
| In-hospital mortality | 2181 (25%) | 180 (14%) | 106 (49%) | 1512 (24%) | 383 (42%) |
| 28-day in-hospital mortality | 1588 (18%) | 106 (8%) | 86 (40%) | 1082 (17%) | 314 (34%) |
| 90-day in-hospital mortality | 2168 (25%) | 180 (14%) | 105 (49%) | 1503 (24%) | 380 (41%) |

Underlined parameters were used for clustering. Data are presented as median [interquartile range], mean (standard error of the mean), or number (%). ^a^Acute physiology score. ^b^Calculated by adding one point for each of the following comorbidities present: AIDS, cardiovascular insufficiency, chronic dialysis, chronic renal insufficiency, cirrhosis, COPD or respiratory insufficiency, diabetes mellitus, hematologic malignancy, immune insufficiency, and metastatic neoplasm. AIDS: acquired immunodeficiency syndrome, APACHE IV: Acute Physiology and Chronic Health Evaluation IV, COVID-19: coronavirus disease 2019, BMI: body mass index, COPD: chronic obstructive pulmonary disease, ARDS: acute respiratory distress syndrome, ICU: intensive care unit.

**Supplementary Table 4: Patient characteristics and outcomes of the non-COVID-19 viral pneumonia cohort.**

| **Parameters obtained within 24 hours of ICU admission** | **All (n=3460)** | **Alpha (n=518, 15%)** | **Beta (n=92, 3%)** | **Gamma (n=2427, 70%)** | **Delta (n=423, 12%)** |
| --- | --- | --- | --- | --- | --- |
| Sex, male | 1823 (53%) | 233 (45%) | 45 (49%) | 1230 (51%) | 315 (74%) |
| BMI, kg/m^2^ | 25.7 [22.6 - 29.7] | 26.2 [22.9 - 31.2] | 27.7 [23.3 - 32.3] | 25.6 [22.6 - 29.5] | 25.6 [22.6 - 28.8] |
| Normal (<25) | 1516 (44%) | 212 (41%) | 34 (37%) | 1080 (44%) | 190 (45%) |
| Overweight (25 to <30) | 1012 (29%) | 140 (27%) | 22 (24%) | 701 (29%) | 149 (35%) |
| Obese Class 1: (30 to < 35) | 464 (13%) | 75 (14%) | 11 (12%) | 330 (14%) | 48 (11%) |
| Class 2: (35 to < 40) | 203 (6%) | 40 (8%) | 13 (14%) | 130 (5%) | 20 (5%) |
| Class 3: (>40) | 148 (4%) | 37 (7%) | 7 (8%) | 98 (4%) | 6 (1%) |
| Age, years | 66 [57 - 74] | 62 [53 - 69] | 71 [64 - 78] | 66 [57 - 74] | 69 [59 - 76] |
| APACHE IV score | 64 [51 - 80] | 45 [36 - 55] | 70 [62 - 87] | 65 [53 - 79] | 86 [73 - 108] |
| APACHE IV APS^a^ score | 50 [39 - 65] | 34 [27 - 43] | 54 [46 - 69] | 51 [41 - 63] | 70 [58 - 90] |
| Aids | 6 (0%) | 1 (0%) | 0 (0%) | 4 (0%) | 1 (0%) |
| Cardiovascular insuffiency | 115 (3%) | 13 (3%) | 10 (11%) | 69 (3%) | 23 (5%) |
| Chronic dialysis | 33 (1%) | 0 (0%) | 10 (11%) | 12 (0%) | 11 (3%) |
| Chronic renal insuffiency | 237 (7%) | 7 (1%) | 52 (57%) | 109 (4%) | 69 (16%) |
| Cirrhosis | 22 (1%) | 2 (0%) | 0 (0%) | 14 (1%) | 6 (1%) |
| COPD | 1551 (45%) | 313 (60%) | 46 (50%) | 1097 (45%) | 95 (22%) |
| Diabetes mellitus | 693 (20%) | 68 (13%) | 52 (57%) | 464 (19%) | 109 (26%) |
| Hematological malignancy | 180 (5%) | 10 (2%) | 7 (8%) | 114 (5%) | 49 (12%) |
| Immunological insufficiency | 640 (18%) | 61 (12%) | 24 (26%) | 447 (18%) | 108 (26%) |
| Metastatic neoplasm | 68 (2%) | 4 (1%) | 0 (0%) | 56 (2%) | 8 (2%) |
| Respiratory insufficiency | 594 (17%) | 143 (28%) | 19 (21%) | 401 (17%) | 31 (7%) |
| Comorbidity index^b^ | 0.88 (0.014) | 0.90 (0.029) | 1.67 (0.083) | 0.84 (0.016) | 0.92 (0.046) |
| Mechanical ventilation | 2449 (71%) | 383 (74%) | 58 (63%) | 1723 (71%) | 285 (67%) |
| PaO_2_ (mmHg) | 74 (64 - 89) | 74 (63 - 86) | 72 (64 - 83) | 82 (69 - 107) | 73 (64 - 88) |
| PaO_2_/FiO_2_ ratio | 167 [112 - 233] | 200 [148 - 258] | 179 [115 - 229] | 160 [108 - 227] | 154 [93 - 237] |
| No ARDS (>300 mmHg) | 316 (9%) | 60 (12%) | 8 (9%) | 195 (8%) | 53 (13%) |
| Mild ARDS (>200 - ≤300 mmHg) | 792 (23%) | 155 (30%) | 24 (26%) | 538 (22%) | 75 (18%) |
| Moderate ARDS (>100 - ≤200 mmHg) | 1315 (38%) | 199 (38%) | 38 (41%) | 932 (38%) | 146 (35%) |
| Severe ARDS (≤100 mmHg) | 623 (18%) | 28 (5%) | 14 (15%) | 473 (19%) | 108 (26%) |
| PaCO_2_, mmHg | 47 [37 - 60] | 54 [42 - 69] | 47 [38 - 60] | 46 [37 - 60] | 40 [31 - 52] |
| Respiratory rate (max), breaths/min | 32 [27 - 39] | 27 [23 - 30] | 26 [23 - 31] | 34 [29 - 40] | 32 [28 - 38] |
| Vasoactive medication | 1541 (45%) | 133 (26%) | 29 (32%) | 1106 (46%) | 273 (65%) |
| Hematocrit (min) | 0.37 [0.32 - 0.41] | 0.40 [0.36 - 0.44] | 0.32 [0.27 - 0.36] | 0.36 [0.32 - 0.40] | 0.34 [0.28 - 0.39] |
| Heart rate (max), beats/min | 116 [101 - 132] | 105 [93 - 119] | 93 [86 - 100] | 120 [105 - 135] | 120 [106 - 135] |
| Mean arterial pressure (min), mmHg | 61 [54 - 69] | 68 [60 - 76] | 63 [54 - 70] | 61 [54 - 68] | 57 [48 - 64] |
| Mean arterial pressure (max), mmHg | 106 [94 - 121] | 110 [98 - 123] | 96 [88 - 112] | 107 [95 - 122] | 98 [86 - 115] |
| Acute renal failure | 402 (12%) | 6 (1%) | 29 (32%) | 199 (8%) | 168 (40%) |
| Creatinine, max, µmol/L | 84 [60 - 127] | 62 [47 - 79] | 224 [149 - 386] | 81 [60 - 112] | 181 [127 - 260] |
| Blood urea nitrogen, mg/dL | 23 [15 - 35] | 16 [12 - 22] | 62 [45 - 83] | 22 [15 - 31] | 46 [33 - 65] |
| Urinary output, L | 1.53 [1.00 - 2.23] | 1.60 [1.06 - 2.29] | 1.50 [0.69 - 2.08] | 1.55 [1.05 - 2.28] | 1.22 [0.60 - 2.03] |
| Bilirubin, µmol/L | 8 [5 - 12] | 6 [5 - 10] | 6 [4 - 8] | 7 [5 - 12] | 15 [9 - 24] |
| Sodium, max, mmol/L | 139 [136 - 142] | 140 [137 - 142] | 139 [137 - 143] | 139 [136 - 142] | 141 [137 - 144] |
| Potassium (max), mmol/L | 4.4 [4.0 - 4.8] | 4.4 [4.0 - 4.7] | 4.9 [4.3 - 5.2] | 4.3 [4.0 - 4.7] | 4.6 [4.1 - 5.2] |
| Glucose (max), mmol/L | 9.9 [7.9 - 12.5] | 8.8 [7.2 - 10.7] | 11.8 [8.4 - 16.1] | 10.0 [8.0 - 12.5] | 11.0 [8.7 - 15.0] |
| pH (min) | 7.36 [7.29 - 7.43] | 7.37 [7.30 - 7.42] | 7.34 [7.26 - 7.40] | 7.37 [7.30 - 7.43] | 7.32 [7.22 - 7.40] |
| Bicarbonate (max), mmol/L | 28 [24 - 32] | 32 [28 - 37] | 27 [23 - 31] | 28 [25 - 32] | 22 [20 - 26] |
| Albumin (min), g/L | 29 [25 - 34] | 35 [32 - 39] | 29 [26 - 33] | 29 [25 - 33] | 26 [22 - 31] |
| White blood cell count (max), x10^9^/L | 11.0 [7.5 - 15.3] | 9.6 [7.0 - 12.7] | 10.7 [7.5 - 14.2] | 11.3 [7.7 - 15.5] | 12.6 [7.5 - 18.3] |
| Thrombocytes (min), x10^9^/L | 198 [143 - 261] | 203 [164 - 268] | 200 [154 - 248] | 202 [149 - 265] | 146 [95 - 223] |
| Temperature, °C | 38.1 [37.4 - 38.8] | 37.7 [37.2 - 38.2] | 37.4 [36.9 - 37.9] | 38.2 [37.6 - 39.0] | 37.8 [37.2 - 38.4] |
| **Outcome parameters** |  |  |  |  |  |
| ICU length-of-stay survivors, days | 4 [2 - 8] | 3 [1 - 6] | 3 [2 - 6] | 4 [2 - 9] | 5 [3 - 13] |
| ICU length-of-stay nonsurvivors, days | 6 [2 - 12] | 6 [2 - 9] | 9 [4 - 15] | 6 [3 - 13] | 5 [1 - 14] |
| ICU mortality | 583 (17%) | 42 (8%) | 19 (21%) | 401 (17%) | 121 (29%) |
| Hospital length-of-stay survivors, days | 11 [7 - 20] | 9 [6 - 14] | 12 [8 - 20] | 12 [7 - 20] | 15 [9 - 30] |
| Hospital length-of-stay nonsurvivors, days | 9 [4 - 17] | 7 [3 - 12] | 10 [5 - 16] | 9 [5 - 18] | 9 [3 - 19] |
| In-hospital mortality | 755 (22%) | 67 (13%) | 30 (33%) | 514 (21%) | 144 (34%) |
| 28-day in-hospital mortality | 676 (20%) | 63 (12%) | 28 (30%) | 459 (19%) | 126 (30%) |
| 90-day in-hospital mortality | 753 (22%) | 67 (13%) | 30 (33%) | 512 (21%) | 144 (34%) |

Underlined parameters were used for clustering. Data are presented as median [interquartile range], mean (standard error of the mean), or number (%). ^a^Acute physiology score. ^b^Calculated by adding one point for each of the following comorbidities present: AIDS, cardiovascular insufficiency, chronic dialysis, chronic renal insufficiency, cirrhosis, COPD or respiratory insufficiency, diabetes mellitus, hematologic malignancy, immune insufficiency, and metastatic neoplasm. AIDS: acquired immunodeficiency syndrome, APACHE IV: Acute Physiology and Chronic Health Evaluation IV, COVID-19: coronavirus disease 2019, BMI: body mass index, COPD: chronic obstructive pulmonary disease, ARDS: acute respiratory distress syndrome, ICU: intensive care unit.

**Supplementary Table 5: Patient characteristics and outcomes of the bacterial pneumonia cohort.**

| **Parameters obtained within 24 hours of ICU admission** | **All (n=19947)** | **Alpha (n=1448, 7%)** | **Beta (n=702, 4%)** | **Gamma (n=13444, 67%)** | **Delta (n=4353, 22%)** |
| --- | --- | --- | --- | --- | --- |
| Sex, male | 12147 (61%) | 871 (60%) | 494 (70%) | 7683 (57%) | 3099 (71%) |
| BMI, kg/m^2^ | 25.2 [22.4 - 29.1] | 26.1 [22.9 - 30.7] | 27.4 [24.1 - 32.0] | 25.0 [22.2 - 28.7] | 25.3 [22.6 - 29.1] |
| Normal (<25) | 9262 (46%) | 559 (39%) | 221 (31%) | 6472 (48%) | 2010 (46%) |
| Overweight (25 to <30) | 5957 (30%) | 426 (29%) | 216 (31%) | 3960 (29%) | 1355 (31%) |
| Obese Class 1: (30 to < 35) | 2406 (12%) | 185 (13%) | 136 (19%) | 1531 (11%) | 554 (13%) |
| Class 2: (35 to < 40) | 947 (5%) | 95 (7%) | 64 (9%) | 616 (5%) | 172 (4%) |
| Class 3: (>40) | 636 (3%) | 90 (6%) | 40 (6%) | 393 (3%) | 113 (3%) |
| Age, years | 69 [60 - 77] | 63 [53 - 71] | 75 [68 - 80] | 69 [60 - 77] | 71 [62 - 78] |
| APACHE IV score | 73 [58 - 91] | 46 [36 - 57] | 71 [62 - 85] | 70 [58 - 85] | 94 [78 - 115] |
| APACHE IV APS^a^ score | 57 [44 - 74] | 34 [27 - 44] | 53 [44 - 65] | 54 [43 - 67] | 76 [61 - 98] |
| Aids | 87 (0%) | 5 (0%) | 2 (0%) | 61 (0%) | 19 (0%) |
| Cardiovascular insuffiency | 877 (4%) | 32 (2%) | 106 (15%) | 489 (4%) | 250 (6%) |
| Chronic dialysis | 244 (1%) | 0 (0%) | 71 (10%) | 75 (1%) | 98 (2%) |
| Chronic renal insuffiency | 1769 (9%) | 8 (1%) | 374 (53%) | 643 (5%) | 744 (17%) |
| Cirrhosis | 286 (1%) | 12 (1%) | 6 (1%) | 125 (1%) | 143 (3%) |
| COPD | 6605 (33%) | 609 (42%) | 294 (42%) | 4697 (35%) | 1005 (23%) |
| Diabetes mellitus | 4186 (21%) | 171 (12%) | 334 (48%) | 2578 (19%) | 1103 (25%) |
| Hematological malignancy | 1030 (5%) | 20 (1%) | 32 (5%) | 630 (5%) | 348 (8%) |
| Immunological insufficiency | 3683 (18%) | 136 (9%) | 157 (22%) | 2414 (18%) | 976 (22%) |
| Metastatic neoplasm | 1054 (5%) | 38 (3%) | 25 (4%) | 765 (6%) | 226 (5%) |
| Respiratory insufficiency | 2336 (12%) | 284 (20%) | 110 (16%) | 1645 (12%) | 297 (7%) |
| Comorbidity index^b^ | 0.84 (0.006) | 0.68 (0.018) | 1.59 (0.038) | 0.79 (0.007) | 0.91 (0.014) |
| Mechanical ventilation | 11321 (57%) | 791 (55%) | 348 (50%) | 7463 (56%) | 2719 (62%) |
| PaO_2_ (mmHg) | 75 (64 - 90) | 74 (63 - 87) | 73 (64 - 87) | 79 (67 - 98) | 74 (63 - 88) |
| PaO_2_/FiO_2_ ratio | 149 [98 - 221] | 180 [127 - 246] | 168 [109 - 238] | 148 [98 - 218] | 138 [91 - 220] |
| No ARDS (>300 mmHg) | 1657 (8%) | 152 (10%) | 69 (10%) | 1034 (8%) | 402 (9%) |
| Mild ARDS (>200 - ≤300 mmHg) | 3617 (18%) | 293 (20%) | 139 (20%) | 2440 (18%) | 745 (17%) |
| Moderate ARDS (>100 - ≤200 mmHg) | 7216 (36%) | 525 (36%) | 227 (32%) | 4960 (37%) | 1504 (35%) |
| Severe ARDS (≤100 mmHg) | 4512 (23%) | 154 (11%) | 119 (17%) | 3059 (23%) | 1180 (27%) |
| PaCO_2_, mmHg | 41 [33 - 52] | 48 [38 - 64] | 42 [35 - 54] | 41 [34 - 52] | 38 [31 - 48] |
| Respiratory rate (max), breaths/min | 32 [27 - 38] | 26 [22 - 30] | 26 [22 - 30] | 33 [28 - 40] | 32 [28 - 38] |
| Vasoactive medication | 9872 (49%) | 305 (21%) | 269 (38%) | 6135 (46%) | 3163 (73%) |
| Hematocrit (min) | 0.34 [0.29 - 0.38] | 0.38 [0.34 - 0.42] | 0.31 [0.27 - 0.36] | 0.34 [0.29 - 0.38] | 0.32 [0.28 - 0.37] |
| Heart rate (max), beats/min | 118 [102 - 135] | 100 [90 - 113] | 89 [80 - 100] | 120 [105 - 137] | 122 [106 - 141] |
| Mean arterial pressure (min), mmHg | 59 [52 - 67] | 69 [61 - 78] | 63 [55 - 72] | 60 [53 - 67] | 55 [47 - 61] |
| Mean arterial pressure (max), mmHg | 100 [89 - 114] | 105 [94 - 118] | 99 [87 - 114] | 101 [90 - 115] | 95 [85 - 109] |
| Acute renal failure | 3650 (18%) | 28 (2%) | 227 (32%) | 1421 (11%) | 1974 (45%) |
| Creatinine, max, µmol/L | 97 [67 - 156] | 64 [50 - 81] | 227 [162 - 354] | 84 [62 - 118] | 192 [134 - 278] |
| Blood urea nitrogen, mg/dL | 28 [18 - 43] | 16 [11 - 22] | 56 [41 - 74] | 25 [17 - 34] | 48 [34 - 68] |
| Urinary output, L | 1.48 [0.92 - 2.23] | 1.71 [1.12 - 2.50] | 1.38 [0.75 - 2.31] | 1.56 [1.04 - 2.30] | 1.01 [0.40 - 1.83] |
| Bilirubin, µmol/L | 10 [6 - 16] | 8 [5 - 13] | 7 [5 - 10] | 9 [6 - 14] | 16 [10 - 27] |
| Sodium, max, mmol/L | 139 [136 - 142] | 140 [138 - 142] | 140 [137 - 143] | 139 [136 - 142] | 140 [137 - 144] |
| Potassium (max), mmol/L | 4.3 [4.0 - 4.8] | 4.2 [3.9 - 4.6] | 4.7 [4.2 - 5.3] | 4.2 [3.9 - 4.7] | 4.6 [4.2 - 5.3] |
| Glucose (max), mmol/L | 9.5 [7.5 - 12.5] | 8.3 [6.8 - 10.3] | 10.2 [7.7 - 13.5] | 9.4 [7.5 - 12.2] | 10.4 [7.9 - 13.9] |
| pH (min) | 7.38 [7.29 - 7.44] | 7.39 [7.32 - 7.44] | 7.35 [7.28 - 7.41] | 7.39 [7.32 - 7.45] | 7.31 [7.20 - 7.40] |
| Bicarbonate (max), mmol/L | 25 [22 - 29] | 30 [26 - 35] | 25 [22 - 29] | 26 [23 - 30] | 22 [19 - 24] |
| Albumin (min), g/L | 26 [22 - 31] | 34 [30 - 38] | 29 [25 - 33] | 26 [22 - 30] | 25 [20 - 29] |
| White blood cell count (max), x10^9^/L | 13.8 [9.4 - 19.6] | 11.4 [8.3 - 15.0] | 12.4 [9.2 - 16.0] | 13.8 [9.6 - 19.4] | 15.5 [9.4 - 22.8] |
| Thrombocytes (min), x10^9^/L | 208 [148 - 285] | 215 [167 - 271] | 211 [164 - 280] | 221 [160 - 301] | 167 [101 - 234] |
| Temperature, °C | 38.0 [37.4 - 38.8] | 37.7 [37.1 - 38.2] | 37.4 [36.9 - 37.9] | 38.2 [37.5 - 38.9] | 37.6 [37.0 - 38.3] |
| **Outcome parameters** |  |  |  |  |  |
| ICU length-of-stay survivors, days | 3 [2 - 7] | 2 [1 - 5] | 3 [1 - 6] | 3 [2 - 7] | 4 [2 - 10] |
| ICU length-of-stay nonsurvivors, days | 3 [1 - 8] | 4 [2 - 9] | 5 [2 - 8] | 4 [2 - 10] | 2 [1 - 6] |
| ICU mortality | 3831 (19%) | 88 (6%) | 106 (15%) | 2122 (16%) | 1515 (35%) |
| Hospital length-of-stay survivors, days | 13 [8 - 22] | 9 [5 - 14] | 12 [7 - 22] | 13 [8 - 22] | 15 [8 - 26] |
| Hospital length-of-stay nonsurvivors, days | 8 [3 - 16] | 7 [3 - 14] | 9 [6 - 20] | 9 [4 - 18] | 6 [2 - 13] |
| In-hospital mortality | 5257 (26%) | 132 (9%) | 183 (26%) | 3090 (23%) | 1852 (43%) |
| 28-day in-hospital mortality | 4705 (24%) | 121 (8%) | 159 (23%) | 2725 (20%) | 1700 (39%) |
| 90-day in-hospital mortality | 5228 (26%) | 131 (9%) | 183 (26%) | 3071 (23%) | 1843 (42%) |

Underlined parameters were used for clustering. Data are presented as median [interquartile range], mean (standard error of the mean), or number (%). ^a^Acute physiology score. ^b^Calculated by adding one point for each of the following comorbidities present: AIDS, cardiovascular insufficiency, chronic dialysis, chronic renal insufficiency, cirrhosis, COPD or respiratory insufficiency, diabetes mellitus, hematologic malignancy, immune insufficiency, and metastatic neoplasm. AIDS: acquired immunodeficiency syndrome, APACHE IV: Acute Physiology and Chronic Health Evaluation IV, COVID-19: coronavirus disease 2019, BMI: body mass index, COPD: chronic obstructive pulmonary disease, ARDS: acute respiratory distress syndrome, ICU: intensive care unit.

**Supplementary Table 6: Patient characteristics and outcomes of the non-pulmonary sepsis cohort.**

| **Parameters obtained within 24 hours of ICU admission** | **All (n=17983)** | **Alpha (n=525, 3%)** | **Beta (n=514, 3%)** | **Gamma (n=7826, 44%)** | **Delta (n=9118, 51%)** |
| --- | --- | --- | --- | --- | --- |
| Sex, male | 10373 (58%) | 294 (56%) | 302 (59%) | 4180 (53%) | 5597 (61%) |
| BMI, kg/m^2^ | 26.2 [23.2 - 30.3] | 25.7 [22.9 - 29.1] | 27.8 [24.4 - 32.7] | 26.3 [23.2 - 30.5] | 26.1 [23.1 - 30.0] |
| Normal (<25) | 7037 (39%) | 213 (41%) | 155 (30%) | 3003 (38%) | 3666 (40%) |
| Overweight (25 to <30) | 5699 (32%) | 168 (32%) | 163 (32%) | 2458 (31%) | 2910 (32%) |
| Obese Class 1: (30 to < 35) | 2631 (15%) | 55 (10%) | 85 (17%) | 1183 (15%) | 1308 (14%) |
| Class 2: (35 to < 40) | 1082 (6%) | 28 (5%) | 50 (10%) | 490 (6%) | 514 (6%) |
| Class 3: (>40) | 821 (5%) | 21 (4%) | 40 (8%) | 404 (5%) | 356 (4%) |
| Age, years | 69 [59 - 77] | 61 [49 - 70] | 73 [65 - 79] | 69 [59 - 76] | 69 [60 - 77] |
| APACHE IV score | 78 [62 - 98] | 42 [32 - 58] | 74 [63 - 88] | 68 [55 - 83] | 90 [74 - 112] |
| APACHE IV APS^a^ score | 62 [47 - 81] | 31 [23 - 43] | 57 [46 - 70] | 52 [41 - 66] | 74 [59 - 95] |
| Aids | 42 (0%) | 3 (1%) | 2 (0%) | 16 (0%) | 21 (0%) |
| Cardiovascular insuffiency | 754 (4%) | 8 (2%) | 59 (11%) | 270 (3%) | 417 (5%) |
| Chronic dialysis | 471 (3%) | 0 (0%) | 84 (16%) | 104 (1%) | 283 (3%) |
| Chronic renal insuffiency | 2389 (13%) | 6 (1%) | 281 (55%) | 541 (7%) | 1561 (17%) |
| Cirrhosis | 542 (3%) | 4 (1%) | 5 (1%) | 86 (1%) | 447 (5%) |
| COPD | 2248 (13%) | 55 (10%) | 111 (22%) | 1094 (14%) | 988 (11%) |
| Diabetes mellitus | 4696 (26%) | 68 (13%) | 258 (50%) | 1815 (23%) | 2555 (28%) |
| Hematological malignancy | 995 (6%) | 31 (6%) | 25 (5%) | 402 (5%) | 537 (6%) |
| Immunological insufficiency | 3467 (19%) | 86 (16%) | 124 (24%) | 1464 (19%) | 1793 (20%) |
| Metastatic neoplasm | 1280 (7%) | 29 (6%) | 30 (6%) | 578 (7%) | 643 (7%) |
| Respiratory insufficiency | 581 (3%) | 24 (5%) | 29 (6%) | 295 (4%) | 233 (3%) |
| Comorbidity index^b^ | 0.70 (0.007) | 0.46 (0.032) | 1.43 (0.049) | 0.60 (0.009) | 0.75 (0.009) |
| Mechanical ventilation | 5323 (30%) | 102 (19%) | 85 (17%) | 1854 (24%) | 3282 (36%) |
| PaO_2_ (mmHg) | 82 (70 - 99) | 85 (72 - 101) | 80 (68 - 96) | 84 (71 - 104) | 80 (69 - 94) |
| PaO_2_/FiO_2_ ratio | 257 [164 - 344] | 318 [204 - 395] | 296 [200 - 371] | 263 [174 - 340] | 248 [154 - 344] |
| No ARDS (>300 mmHg) | 5352 (30%) | 164 (31%) | 173 (34%) | 2253 (29%) | 2762 (30%) |
| Mild ARDS (>200 - ≤300 mmHg) | 4053 (23%) | 62 (12%) | 101 (20%) | 1905 (24%) | 1985 (22%) |
| Moderate ARDS (>100 - ≤200 mmHg) | 3647 (20%) | 60 (11%) | 69 (13%) | 1459 (19%) | 2059 (23%) |
| Severe ARDS (≤100 mmHg) | 1366 (8%) | 11 (2%) | 25 (5%) | 513 (7%) | 817 (9%) |
| PaCO_2_, mmHg | 34 [29 - 40] | 36 [32 - 43] | 36 [30 - 42] | 35 [30 - 41] | 32 [27 - 39] |
| Respiratory rate (max), breaths/min | 30 [25 - 35] | 22 [20 - 26] | 23 [20 - 26] | 30 [26 - 36] | 30 [25 - 36] |
| Vasoactive medication | 12140 (68%) | 159 (30%) | 265 (52%) | 4563 (58%) | 7153 (78%) |
| Hematocrit (min) | 0.31 [0.27 - 0.35] | 0.33 [0.28 - 0.37] | 0.29 [0.26 - 0.33] | 0.31 [0.27 - 0.35] | 0.31 [0.26 - 0.35] |
| Heart rate (max), beats/min | 116 [100 - 135] | 94 [83 - 106] | 85 [76 - 96] | 118 [104 - 135] | 119 [102 - 137] |
| Mean arterial pressure (min), mmHg | 55 [48 - 62] | 63 [57 - 72] | 59 [52 - 66] | 57 [50 - 64] | 53 [45 - 60] |
| Mean arterial pressure (max), mmHg | 94 [84 - 106] | 97 [86 - 110] | 92 [82 - 103] | 96 [86 - 108] | 92 [82 - 104] |
| Acute renal failure | 6283 (35%) | 27 (5%) | 239 (46%) | 1353 (17%) | 4664 (51%) |
| Creatinine, max, µmol/L | 146 [93 - 237] | 72 [57 - 87] | 304 [192 - 520] | 101 [73 - 143] | 205 [142 - 303] |
| Blood urea nitrogen, mg/dL | 35 [22 - 54] | 16 [11 - 20] | 61 [44 - 84] | 25 [17 - 36] | 46 [32 - 67] |
| Urinary output, L | 1.35 [0.66 - 2.20] | 1.90 [1.21 - 2.87] | 1.17 [0.37 - 2.05] | 1.65 [1.06 - 2.48] | 1.00 [0.34 - 1.90] |
| Bilirubin, µmol/L | 14 [8 - 27] | 11 [7 - 17] | 7 [5 - 11] | 11 [7 - 17] | 21 [11 - 44] |
| Sodium, max, mmol/L | 139 [136 - 142] | 140 [137 - 142] | 138 [136 - 141] | 138 [135 - 141] | 139 [136 - 143] |
| Potassium (max), mmol/L | 4.3 [4.0 - 4.9] | 4.0 [3.7 - 4.3] | 4.6 [4.1 - 5.3] | 4.2 [3.8 - 4.5] | 4.6 [4.1 - 5.2] |
| Glucose (max), mmol/L | 9.0 [7.1 - 12.1] | 7.2 [6.0 - 9.0] | 9.1 [7.1 - 12.3] | 8.7 [7.0 - 11.4] | 9.5 [7.2 - 13.0] |
| pH (min) | 7.38 [7.30 - 7.44] | 7.42 [7.38 - 7.45] | 7.38 [7.31 - 7.43] | 7.41 [7.37 - 7.46] | 7.34 [7.25 - 7.41] |
| Bicarbonate (max), mmol/L | 22 [19 - 24] | 25 [22 - 28] | 23 [20 - 26] | 24 [21 - 26] | 20 [17 - 22] |
| Albumin (min), g/L | 24 [20 - 28] | 32 [28 - 35] | 26 [22 - 30] | 24 [20 - 28] | 23 [19 - 27] |
| White blood cell count (max), x10^9^/L | 15.9 [10.2 - 23.4] | 10.0 [6.5 - 14.3] | 13.1 [8.8 - 18.4] | 15.1 [10.1 - 21.6] | 17.4 [11.0 - 25.8] |
| Thrombocytes (min), x10^9^/L | 169 [104 - 254] | 178 [119 - 245] | 208 [142 - 289] | 197 [134 - 287] | 140 [79 - 219] |
| Temperature, °C | 38.0 [37.3 - 38.9] | 37.8 [37.2 - 38.5] | 37.4 [36.8 - 37.9] | 38.5 [37.8 - 39.4] | 37.6 [37.0 - 38.4] |
| **Outcome parameters** |  |  |  |  |  |
| ICU length-of-stay survivors, days | 2 [1 - 5] | 1 [1 - 3] | 2 [1 - 4] | 2 [1 - 4] | 3 [2 - 6] |
| ICU length-of-stay nonsurvivors, days | 2 [1 - 5] | 2 [1 - 11] | 3 [1 - 6] | 3 [1 - 8] | 2 [1 - 4] |
| ICU mortality | 3250 (18%) | 15 (3%) | 35 (7%) | 667 (9%) | 2533 (28%) |
| Hospital length-of-stay survivors, days | 13 [7 - 24] | 7 [4 - 15] | 14 [8 - 24] | 12 [7 - 25] | 14 [7 - 25] |
| Hospital length-of-stay nonsurvivors, days | 7 [2 - 17] | 11 [4 - 18] | 14 [7 - 24] | 10 [4 - 22] | 5 [2 - 14] |
| In-hospital mortality | 4456 (25%) | 27 (5%) | 98 (19%) | 1125 (14%) | 3206 (35%) |
| 28-day in-hospital mortality | 3932 (22%) | 24 (5%) | 79 (15%) | 936 (12%) | 2893 (32%) |
| 90-day in-hospital mortality | 4407 (25%) | 26 (5%) | 97 (19%) | 1106 (14%) | 3178 (35%) |

Underlined parameters were used for clustering. Data are presented as median [interquartile range], mean (standard error of the mean), or number (%). ^a^Acute physiology score. ^b^Calculated by adding one point for each of the following comorbidities present: AIDS, cardiovascular insufficiency, chronic dialysis, chronic renal insufficiency, cirrhosis, COPD or respiratory insufficiency, diabetes mellitus, hematologic malignancy, immune insufficiency, and metastatic neoplasm. AIDS: acquired immunodeficiency syndrome, APACHE IV: Acute Physiology and Chronic Health Evaluation IV, COVID-19: coronavirus disease 2019, BMI: body mass index, COPD: chronic obstructive pulmonary disease, ARDS: acute respiratory distress syndrome, ICU: intensive care unit.
